# Fish-brine Odor

**Published:** 1888-04

**Authors:** E. W. Berridge

**Affiliations:** London


### FISH-BRINE ODOR.

E. W. BERRIDGE, M. D., LONDON.

Dr. G. H. Clark's valuable *Repertory of Aural Discharges* has suggested the following comparison:

*Calcarea* has—Oozing of fluid from rectum, smelling like herring-brine. *Guiding Symptoms*, p. 167.

*Graphites* has—The scab of an ulcer, smells like herring-brine. *Encyclopædia*, Symptom 1038.

Medorrhinum has—Oozing of moisture from anus, fetid like fish-brine.

Sanicula has—Vaginal discharge after coitus, smelling like fish-brine.

Tellurium has—Discharge from ears smelling like fish-brine.

Are there any others?

Under Pterygium, in addition to those he names, my *Eye Repertory* gives *Arg-n.*, *Apis*, *Arsen.*, *Cannab.*, *Chelid.*, *Euphr.*, (*Formica*), *Laches.*, *Nux-m.*, *Puls.*, *Ratanh*, *Ruta*, *Spig.* (*Trombid*).

---

CHELIDONIUM.—Dry cough through the day with pain and stitches in the *right* side, with severe *hoarseness* each evening at *five o'clock*, so that her voice could scarcely be heard.—C. CARLETON SMITH.

